# A Wireless Indoor Environmental Quality Logger Processing the Indoor Global Comfort Index

**DOI:** 10.3390/s22072558

**Published:** 2022-03-27

**Authors:** Stefano Riffelli

**Affiliations:** Department of Applied and Pure Sciences (DiSPeA), University of Urbino Carlo Bo, 61029 Urbino, Italy; s.riffelli@campus.uniurb.it

**Keywords:** indoor environmental quality (IEQ) logger, Raspberry Pi, sensors, Internet of Things (IoT) thermal comfort, indoor air quality (IAQ), visual comfort, acoustic comfort, indoor global comfort index (IGCI), MATLAB, SPSS

## Abstract

Indoor environmental quality (IEQ) has a high-level of impact on one’s health and productivity. It is widely accepted that IEQ is composed of four categories: thermal comfort, indoor air quality (IAQ), visual comfort, and acoustic comfort. The main physical parameters that primarily represent these comfort categories can be monitored using sensors. To this purpose, the article proposes a wireless indoor environmental quality logger. In the literature, global comfort indices are often assessed objectively (using sensors) or subjectively (through surveys). This study adopts an integrated approach that calculates a predicted indoor global comfort index (P-IGCI) using sensor data and estimates a real perceived indoor global comfort index (RP-IGCI) based on questionnaires. Among the 19 different tested algorithms, the stepwise multiple linear regression model minimized the distance between the two comfort indices. In the case study involving a university classroom setting—thermal comfort and indoor air quality were identified as the most relevant IEQ elements from a subjective point of view. The model also confirms this findings from an objective perspective since temperature and CO_2_ merge as the measured physical parameters with the most impacts on overall comfort.

## 1. Introduction

Building sector stakeholders have become increasingly involved in systems that are capable of acquiring, storing, and analysing building data through the Internet of Things (IoT) [1]. The high flexibility of new embedded systems allows their application in fields, such as indoor environmental quality (IEQ) management and energy savings. It is well-known that people spend many hours indoors. According to a study conducted by the World Health Organization [2], populations in developed countries spends approximately 90% of their time in indoor environments, such as homes, offices, schools, etc. Comfort in these types of environments is becoming increasingly important as there is now widespread evidence that it impacts health, well-being and productivity [3,4,5]. It is widely accepted that a user’s comfort, or indoor environmental quality (IEQ) [6,7,8], consists of four core parameters, also known as IEQ elements, IEQ factors, or IEQ categories. These are *thermal comfort* [9,10,11,12,13], *indoor air quality (IAQ)* [14,15,16,17], *acoustic comfort* [18,19,20], and *visual comfort* [21,22,23,24,25,26]. Achieving high IEQ levels could prevent the occurrence of sick building syndrome (SBS) [27,28,29], building-related diseases, multiple chemical sensitivities (MCS), and other unrecognized controversial disorders [30]. Several studies [31,32,33,34,35,36,37,38,39,40,41,42,43,44,45,46] have identified weightings of these IEQ factors and/or have proposed overall comfort indices. These global comfort indices (GCIs) and their categories are often assessed either objectively (using sensors) or subjectively (through surveys) [20,47,48,49,50]. Assessing the impact of each IEQ category on overall comfort is challenging for multiple reasons [51]. First, the physical environmental factors (such as CO_2_ concentration, noise level, temperature, and illuminance) influence the corresponding comfort category, as well as the other categories, although to a lesser degree [52]. The IEQ factor weightings largely depend on the occupant’s expectations and satisfaction toward the corresponding factors [53]. For example, if occupants are not satisfied with acoustic comfort, this category becomes more relevant. The IEQ category weightings also depend on building type (e.g., commercial, residential, and educational buildings), other building-related factors (e.g., geographic location, ventilation system, public or private property, new or existing) [51], as well as seasonal changes and external climate [54]. Finally, IEQ (such as the IEQ productivity belief) is also affected by attitudinal and behavioural factors, social influence factors, and demographic aspects of the building occupants (such as gender, age, and cultural difference) [55]. Different methods have attempted to set these weightings but have led to different results [51].

This research aimed to identify a methodology to predict perceived comfort by directly measuring physical parameters in a given indoor environment. A wireless IEQ logger system was designed to this aim, comprising hardware, software components, and a data analysis. The idea was to assemble a hardware system that is expandable and has the necessary resources for autonomous data processing. For this reason, a microprocessor-based embedded system (Raspberry Pi [56]) was chosen, rather than a microcontroller-based one (such as Arduino [57]). For the sake of simplicity, practicality, and compactness, some environmental kits were considered, rather than individual sensors to be connected directly to the board. These kits were Metriful [58], OKdo air quality kit [59], and Enviro+ [60]. Metriful uses the MS430 all-in-one sensor. This is a very cheap sensor (it costs about EUR 40) but it is currently out of stock on the market. The OKdo air quality kit adopts a “Base HAT” to connect the Aosong AM2302 temperature and humidity sensor and the Sensirion SGP30 sensor to measure volatile organic compounds (VOCs) and eCO_2_ (carbon dioxide equivalent). This complete kit costs about EUR 50. Enviro+ (by Pimoroni) was the final choice; it is currently available on the market (for about EUR 50) and it is one of the most complete models (continue reading for details). IEQ data collection/processing is divided into multiple steps. *Implementation*: the IEQ logger was built, adopting the DIY philosophy (in Section 2). The main hardware components are an IEQ control unit and sensors measuring physical quantities associated with indoor environmental quality (i.e., thermal comfort, indoor air quality (IAQ), visual comfort, and acoustic comfort). The software system includes the sensor libraries and control unit, a database for data collection, online questionnaires, and a graphical web interface. *Deployment*: the IEQ logger was positioned in a university classroom and registered 29 university lectures over the course of 3 months (in Section 3.1). *Data collection*: physical parameters measured by the sensors (objective data) and questionnaires filled by students (subjective data) were collected and stored in a MySQL database (in Section 3.2). *Data analysis*: after reviewing methods frequently adopted in the field (in Section 3.3), regression analysis was performed to correlate the overall comfort reported in the questionnaires with the individual comfort categories and the physical parameters measured by sensors (in Section 4.1). *Model building and characterization*: 19 different algorithms were tested in order to identify the method that minimized the distance between a predicted and a perceived comfort index (in Section 4.2). The first index was calculated from sensor data, while the second was based on questionnaires. The obtained model estimates the perceived comfort index based on measured physical quantities.

## 2. IEQ Logger Hardware and Software

This section describes the hardware needed to build the complete IEQ logger system and the software architecture for proper data acquisition and storage provided by the sensors. Thermal comfort was measured with air temperature (in degrees Celsius) and relative humidity (as a percentage). IAQ was measured with CO_2_ concentration (in parts per million). Visual comfort was measured with illuminance (in Lux). Acoustic comfort was measured with noise level (in A-weighted decibels). Table 1 summarizes the comfort categories with all corresponding measured physical parameters and units.

The type of thermal comfort assessment depends on the adopted approach. The first approach consists of determining the predicted mean vote (PMV) and predicted percentage dissatisfied (PPD) indices according to the ISO 7730 standards [9] that define them. The determination of the PMV and PPD indices is carried out through specific professional instrumentation, such as microclimatic control units based on “spot measurements”. The instrumentation must conform to the requirements specified in the ISO 7726 standard [61]. The advantage of this methodology is the high measurement accuracy. The PMV method requires input clothing insulation (CI), metabolic rate (MR), air speed (AS), mean radiant temperature (MRT), air temperature (AT), and relative humidity (RH). Of these parameters, the last four are directly measurable. The second approach is to determine the behaviour of parameters, such as AS, MRT, AT, and RH through a data logger and “frequent measurements”. In our case study, the room analysis and the building typology allows neglecting the AS, which is definitely lower than 0.2 m/s. The MRT measures the average temperature of the surfaces surrounding a particular point with which thermal radiation is exchanged. The knowledge of surface geometries needed to predict MRT is complex, particularly in elaborate spaces. The evaluation of the MRT, whose methodology is also defined in the ISO 7726 standard [61], is neither immediate nor straightforward. The standard considers three measurement methods (globe thermometer, two-sphere radiometer, and constant air temperature sensor) and two calculation methods (view factors and radiant plane temperatures) [62]. Currently, some instruments for measuring MRT are available on the market. The most widely used, and the least expensive, is undoubtedly the globe thermometer, but it has several disadvantages:

- High response time (which leads to problems when numerous measurements are performed);
- Overestimating radiant contributions due to horizontal surfaces (ceiling and floor), due to its (perfectly) spherical shape;
- Not allowing the radiant temperature asymmetry calculation in moderate environments;
- Complex interfacing to the embedded systems (such as Raspberry Pi [56]);
- The required instrumentation would not be suitable for the environment in question (a classroom full of students) but rather for a “controlled” environment or a laboratory.

Air temperature is the most influential and easily measured objective datum. Furthermore, by simulating different scenarios with the CBE Thermal Comfort Tool [63], it was possible to carry out several tests concerning the ASHRAE-55 [12] and EN-16798 [7] standards (both with the “PMV” and “adaptive” methods). Given the few differences (as far as this case study is concerned), and the sensors on the market, it was decided to follow the second approach and investigate only the direct measurements of AT and RH. In summary, in this context, the chosen methodology for thermal comfort produces less precise measurements but is undoubtedly cheaper, simpler, more compact, and better in terms of interfacing.

The human ear is most sensitive to sound at frequencies between 1 and 4 kHz [64]. It reaches its maximum sensitivity in the 800 to 2000 Hz frequency range, and it also strongly attenuates sounds below 400 Hz. Please note that the noise level is measured in dBA to take into account the human ear sensitivity.

## 2.1. Hardware Implementation

**Figure 1:**
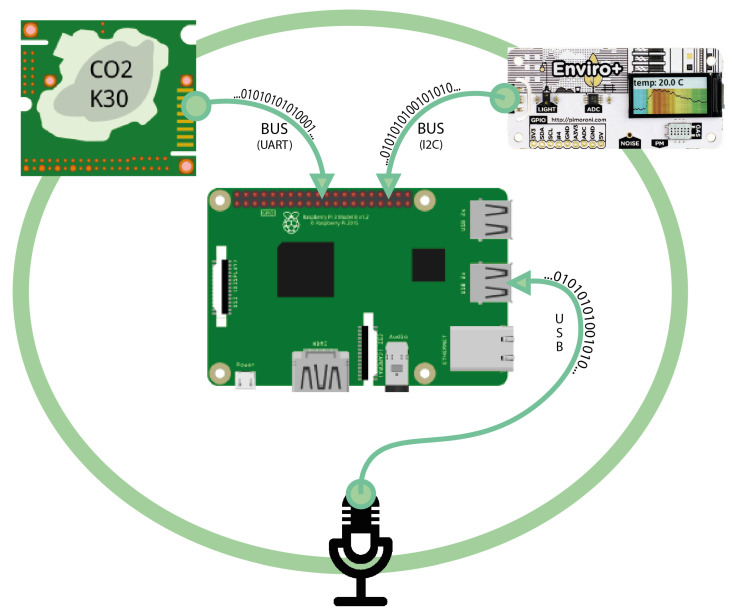
Wireless IEQ logger: hardware architecture.

Raspberry Pi 3 Model B+ [65], Enviro+ by Pimoroni [60], K30 (CO_2_ sensor) [66], and the USB omnidirectional condenser microphone were adopted as part of the hardware development of the IEQ logger. The hardware architecture is shown in Figure 1.

**Figure 2:**
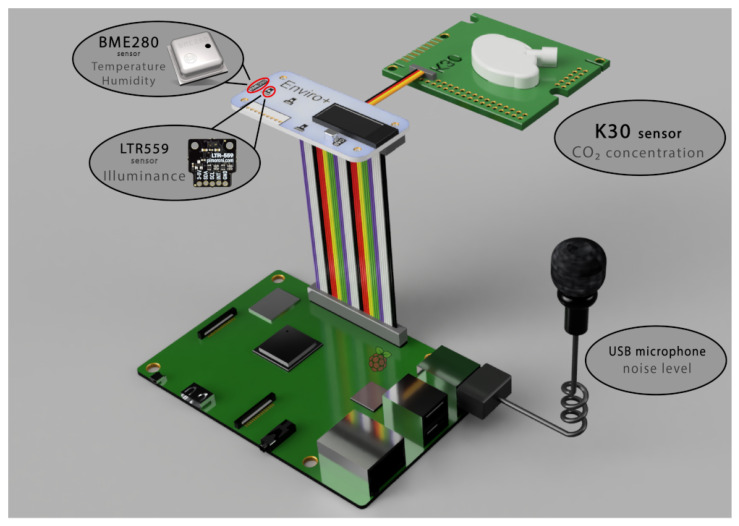
Sensors adopted by the system.

In addition, other hardware was adopted, such as a “40-Pin cable” for the connection between Raspberry Pi and Enviro+, the “GPIO Pin header”, to split the necessary wires for the K-30 sensor connection, and an external box (ABS case). A common micro USB power supply with an output voltage of 5 V and a maximum current of 3 A was employed. Other hardware was exclusively required for sensor calibration and will be described later. Figure 2 shows the sensors adopted by the system and their connections.

Table 2 summarizes the sensors used to monitor the considered physical parameters, while Table 3 presents the technical features of the sensors.

For more technical features and details, please refer to the corresponding datasheet for BME280 [67], LTR-559 [68], and K-30 [66]. The Enviro+ board includes the following sensors: BME280 (temperature, pressure, humidity sensor), LTR-559 (light and proximity sensor), MICS6814 (analogue gas sensor), and SPH0645LM4H-B (MEMS microphone). The board also contains an ADS1015 analogue to the digital converter (ADC), 0.96″ colour LCD (16 mm × 8 mm), and a connector for the particulate matter (PM5003) sensor. Finally, other features include a power supply of 5 V, a 40-pin header Raspberry Pi model compatible (uses 16 GPIO pins), a communication interface I^2^C, and dimensions of 65 mm × 30 mm × 8.5 mm. For more details, please refer to the official website [60] and pinout [69]. The Enviro+ board by Pimoroni was mainly used to detect air temperature, relative humidity, and light level (thanks to the BME280 and LTR-559 sensors).

Currently, the microelectromechanical systems (MEMS) microphone does not have full support, as the official Pimoroni website reports [70]). Furthermore, running several tests with the available libraries, the noise detection range was reduced to a few meters and, therefore, it was not very suitable for our purpose. For these reasons, a USB omnidirectional condenser microphone (by Gyvazla brand) was chosen to detect ambient noise. This is a low-cost microphone (EUR 10) that had good features for our study. The MICS6814 analog gas sensor [71] detects many different types of gases, such as carbon monoxide CO, nitrogen dioxide NO_2_, ethanol C_2_H_5_OH, hydrogen H_2_, ammonia NH_3_, methane CH_4_, propane C_3_H_8_, and isobutane C_4_H_10_. However, this sensor does not detect carbon dioxide CO_2_. For this purpose, the K-30 sensor was added to the system. This sensor measures real (not equivalent) CO_2_. It is a mid-to-high-end sensor with a good price–performance ratio (it costs approximately EUR 60).

**Figure 3:**
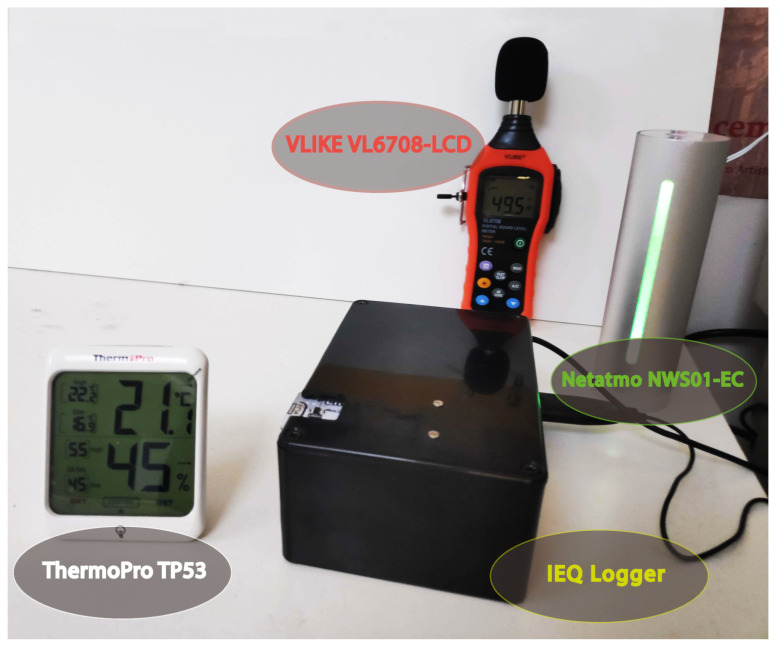
Instruments during calibration and testing phases.

For the calibration and testing phases of the different sensors, the following instruments were adopted: Sound Level Meter, VLIKE VL6708-LCD (for USB microphone calibration), Netatmo NWS01-EC (for K30 CO_2_ sensor calibration), ThermoPro TP53 (for temperature and humidity sensor calibration BME280), and a consumer-grade smartphone with the corresponding app for the brightness sensor. Figure 3 shows the instruments in operation during the calibration and testing phases.

A smartphone app, i.e., “Lux light meter”, was used for Lux calibration, measured by the LTR-559 sensor, and the “shift” was corrected via software. The sensor was tested with different light types (with a bulb dimmable in light colour and intensity). The calibration of the temperature (in degrees Celsius) and the humidity (in percent), measured by the BME280 sensor, was performed via software. The sensor was tested in a room with a heating, ventilation, and air conditioning (HVAC) system (in order to obtain different temperature/humidity conditions). The readings were compared with the values shown on the ThermoPro TP53 display. Calibration of the dBA measured by the USB omnidirectional condenser microphone was performed via software. The microphone was placed close to the sound level meter. A sound generator (at different frequencies) was used to obtain different noise levels to compare with the values of the VLIKE VL6708 sound level meter (displayed on the LCD). Lastly, the calibration of the CO_2_ concentration was conducted via hardware. The sensor was placed in an outdoor environment (in fresh air corresponding to 400 ppm), and Din1 was connected to the ground for at least 8 s (as instructed in the datasheet [66]). In this way, the internal calibration code background calibration (bCAL) was executed. Then, simply by spending some time in a room, it was possible to compare the values between the K-30 sensor and Netatmo NWS01-EC. All sensors were tested in a value range suitable for an indoor environment under non-extreme conditions. For technical details, specifications and more information on these devices, please visit the corresponding web pages for VLIKE VL6708-LCD [72], Netatmo NWS01-EC [73], and ThermoPro TP53 [74].

## 2.2. Software Implementation

**Figure 4:**
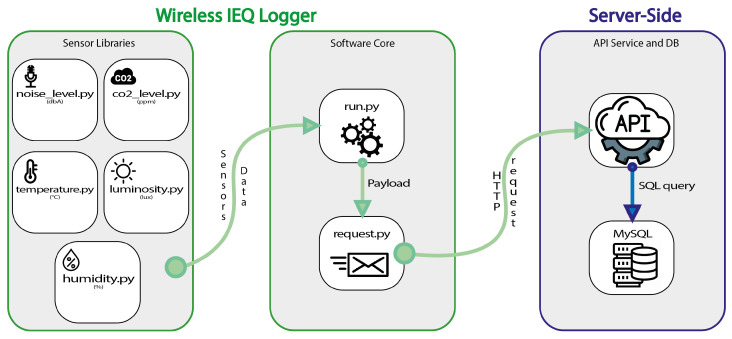
Wireless IEQ logger: software architecture.

In structural terms, the software implemented for the IEQ logger system can be divided into three macroblocks: (i) Sensor libraries, (ii) software core, (iii) API service and database. The software architecture is shown in Figure 4.

The *sensor libraries* contain all of the adopted libraries and are implemented in Python programming language. Each library defines the methodologies for measurements from the corresponding sensors as shown in Table 4:

The *software core* represents the central part of the system. Inside it, run.py is the main Python script that calls up the previous sensor libraries. The purpose of run.py is to obtain the reliable value of each sensor from the various libraries and generate a “payload” (in JSON format). In addition to the sensor parameters, the username and password were added at the payload beginning to perform operations on the online application programming interface (API). For security reasons, authentication was server-sided, and it was implemented in PHP scripting language. Furthermore, the run.py file uses the methods contained in the request.py file. This last file has the only aim of obtaining the “payload” as input and sending an HTTP request to the API server located on the website (which will be discussed later).

The *server-side* includes the database and the required API services for interfacing. Each module provides a different service, such as generating a new record, obtaining one or more records from the database, and so on. The database is implemented in MySQL and mainly consists of records from questionnaires and measurements of all sensors stored in two different tables.

**Figure 5:**
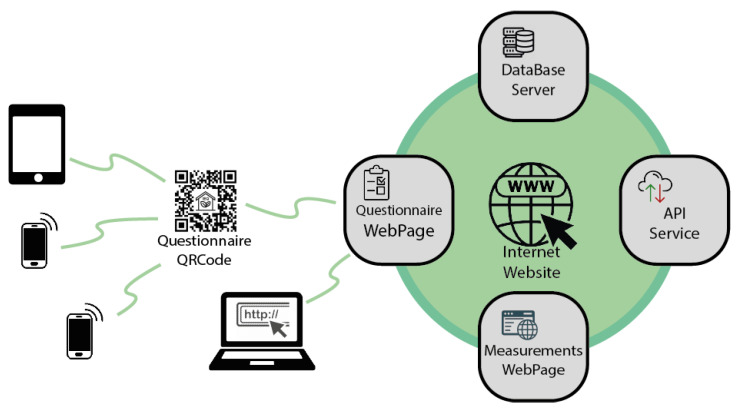
Website components.

A website was developed to implement the questionnaire and to allow the link to be reached via a QR code (for quick access from smartphones and tablets), collect questionnaire information into the database, and report the acquired measures in a user-friendly layout. The main components of the website are summarized in Figure 5.

**Figure 6:**
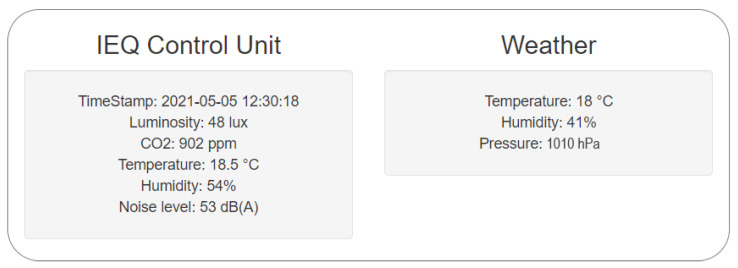
A web page layout example of measurements.

A measurements web page layout example is presented in Figure 6, while the questionnaire structure is described in Section 3.2.

To simultaneously provide information about the status of the outdoor conditions, such as temperature, humidity, and pressure, a weather section was added (thanks to the OpenWeatherMap API service [75]).

## 3. Case Study and Methods

### 3.1. Deployment

A wireless IEQ logger was installed in a classroom of the university, located on the second and top floor of “Collegio Raffaello” building. This classroom has an area of about 70 m^2^ (8.2 m × 8.5 m) and an average height of 2.8 m. Therefore the total volume is about 195 m^3^. Approximately 10 students (at minimum) usually occupy this classroom, to a maximum of 30 (due to the COVID-19 pandemic), depending on the university course. There are two windows in total; they are located at the bottom of the classroom, in the wall facing south-east. The total window surface is 4 m^2^. Four natural light neon lamps provide artificial light. There are no HVAC or mechanical ventilation systems in the classroom. Two doors are 1.1 m wide and 2.1 m high for a total area of 4.6 m^2^. The only airflow is through these doors and the windows (normally closed during lessons). Heating is provided by three cast iron radiators of 0.1 m^3^ each. The system was installed at a height of 1.6 meters from the floor and approximately halfway up one side of the classroom. This height was considered as a reasonable average to measure the CO_2_ (which stratifies downwards), the brightness (considering blackboard, windows, and eye-level), and the noise perceived by the students. The IEQ logger placement on an internal wall was chosen for practical reasons and to find a position that did not create an obstacle for people (both for the passage and view). The IEQ logger position may not be the best for thermal comfort. However, no appreciable differences compared to the classroom center were observed during testing and calibration. According to the tests carried out before and during the calibration phases, this installation on the internal wall still guaranteed good temperature measurements. The position is the most suitable for the other three categories of comfort. The chosen position met the following requirements:

- It was sufficiently far from radiators or windows, allowing for correct temperature and humidity measurement;
- It was at a medium height, in order to correctly measure the CO_2_ concentration (corresponding approximately to the height of the air inhaled by people);
- It was in the middle of the side, because it was optimal for the perceived noise level (not too close to the teacher’s voice) and to detect both the artificial light (from neon) and natural light (from the windows at the bottom of the classroom);
- It was not too far from the wireless repeater (to ensure a good wireless signal).

**Figure 7:**
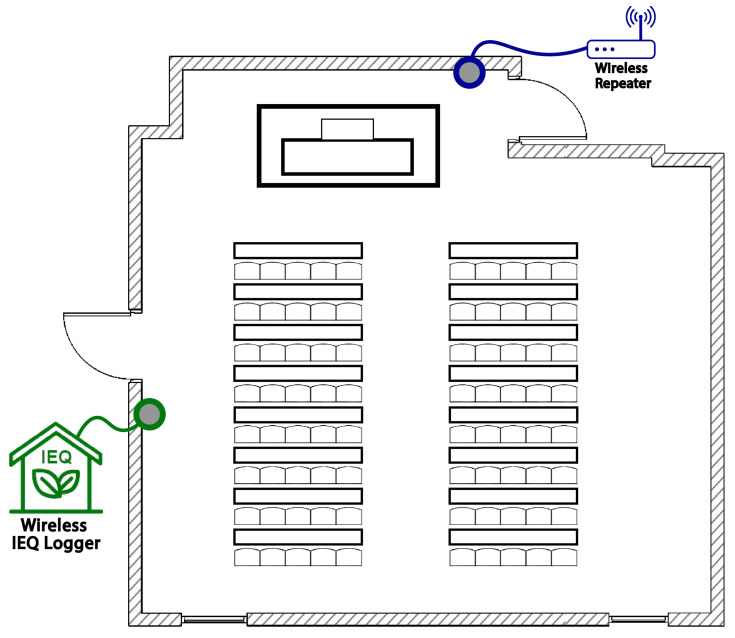
IEQ placement on the university classroom plan.

**Figure 8:**
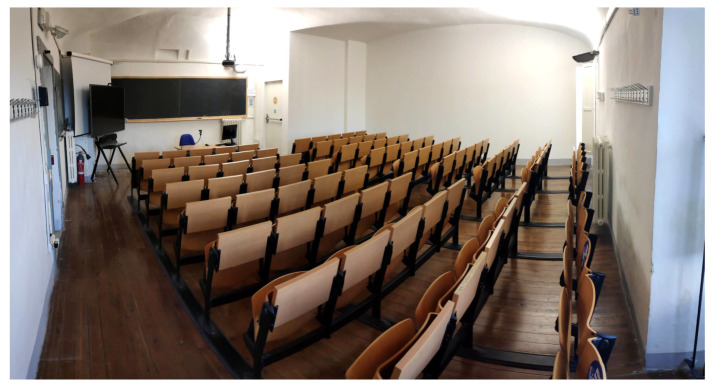
A picture of the examined university classroom.

**Figure 9:**
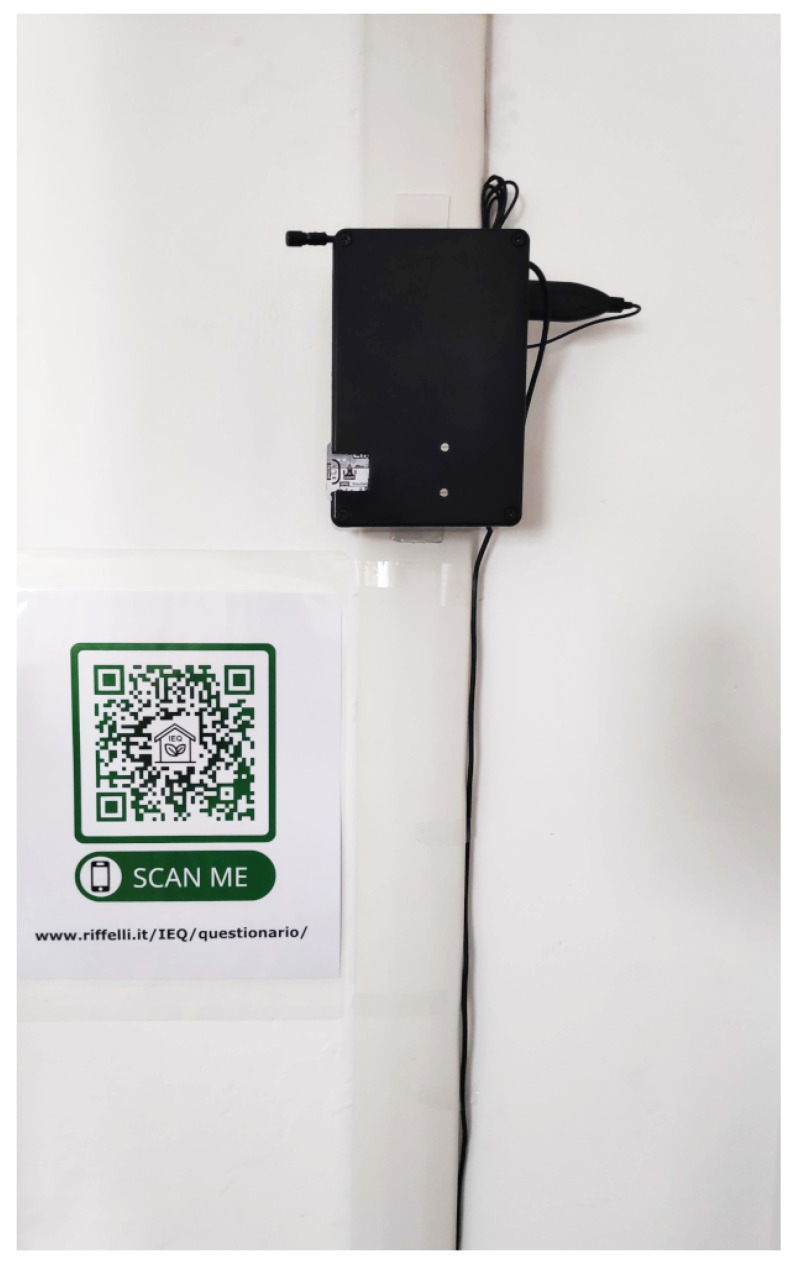
Wireless IEQ logger: system installation in the classroom.

Eventually, the position was also comfortable, being close to a socket. Figure 7 illustrates the university classroom plan with the wireless IEQ logger and wireless repeater locations. A picture of the classroom is shown in Figure 8 in order to provide a better idea of the environment. Finally, the wireless IEQ logger installation in the classroom is shown in Figure 9.

### 3.2. Data Collection

**Figure 10:**
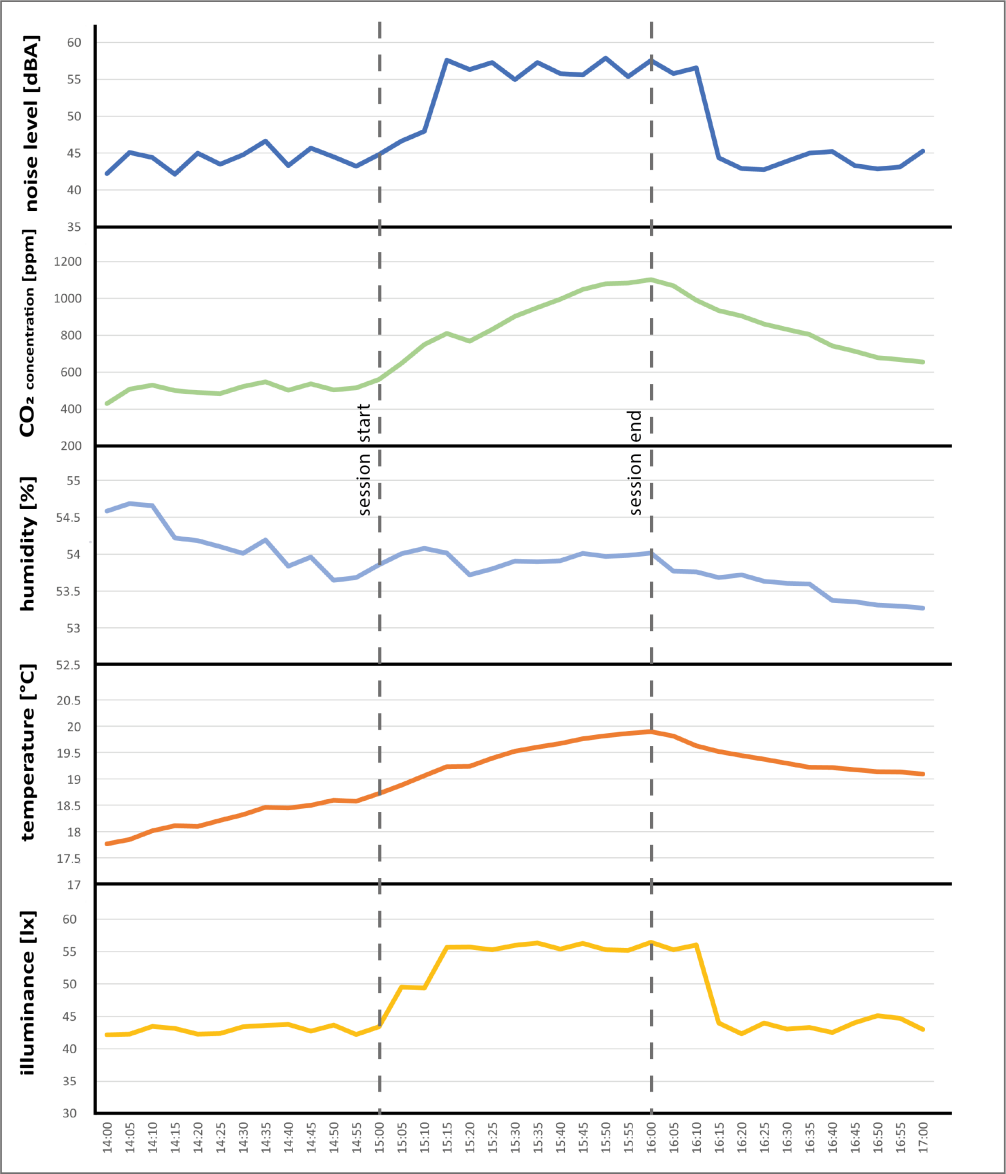
Measured physical parameters in a session example.

The study was carried out during the months of March, April, and May 2021. More precisely, 85 complete questionnaires were collected from 3 March to 28 May and were grouped into 29 sessions. A session was defined as a classroom lesson unit, typically one hour long. Moreover, 10% of the sessions, with either a few or only one questionnaire carried out improperly, were not included in the analysis described in the following section. Environmental parameter data provided by the sensors were recorded every 5 min. This time interval is adequate in order to avoid a data overload on the database, and it is adjustable. In this way, within one-hour sessions, there are 12 different recordings for each measured parameter. Figure 10 shows the parameters measured in a typical session. It is evident how each factor is affected by the occupants.

Sensor data were directly uploaded to the online database via a wireless connection. In case of connection problems, the data were locally stored (in a file on a microSD) and uploaded to the database as soon as the internet connection was back. On the other hand, subjective data were collected by accessing the following online questionnaire. The questionnaire was divided into two parts: “Basic information” and “Comfort categories in the last hour”. The questionnaire was drawn for a university classroom [48], adopting the post-occupancy evaluation (POE) method [47]. The response range for the comfort sensation was from 1 to 5 [20], where 1 is “very poor” and 5 is “very good”. All participation was voluntary at the end of the lecture hour (session).

**IEQ QUESTIONNAIRE**

BASIC INFORMATION:

Gender: Male | Female | Not declared

Age: 19 | 20 | 21 | 22 | 23 | 24 | 25 | 26 | 27 | 28 | 29 | 30+

How do you rate **global** comfort in the classroom?

VERY POOR 1 | 2 | 3 | 4 | 5 VERY GOOD

COMFORT CATEGORIES IN THE LAST HOUR:

How do you rate **thermal** comfort in the classroom?

VERY POOR 1 | 2 | 3 | 4 | 5 VERY GOOD

How do you rate the **air quality** in the classroom?

VERY POOR 1 | 2 | 3 | 4 | 5 VERY GOOD

How do you rate **visual** comfort in the classroom?

VERY POOR 1 | 2 | 3 | 4 | 5 VERY GOOD

How do you rate **acoustic** comfort in the classroom?

VERY POOR 1 | 2 | 3 | 4 | 5 VERY GOOD

### 3.3. Methods

A multiple linear regression (MLR) analysis is a technique used to analyze the linear relationship between a dependent variable (output/response variable) and two or more independent variables (inputs/predictors). The MLR can be adopted for two purposes: *explanatory*; that is, understanding and weighing the effects of independent variables on the dependent variable as a function of a given theoretical model; and *predictive/estimative*, to identify a linear combination of independent variables to best predict/estimate the assumed value by the dependent variable.

In previous studies on comfort indices, MLR analyses were performed, or weights were assigned to different comfort categories in order to provide an indoor global comfort index (IGCI) [31,33,34,41,42,76,77]. From these studies, it is possible to generalize the formula for an indoor global comfort index (IGCI):

(1) $$\begin{array}{c} IGCI=c+W_{1}I_{1}+W_{2}I_{2}+\ldots +W_{n}I_{n} \end{array}$$

where *c* is the constant or intercept (which is zero when passing through the origin), *I* are the different comfort categories (expressed as indices of one or more physical parameters or as satisfaction/dissatisfaction indices), *W* are the corresponding weights, and *n* are the index numbers taken into account. In this case study, *n* = 4 when MLR is applied for subjective data (corresponding to the four comfort categories) while *n* = 5 when MLR is applied for objective data (corresponding to the five measured parameters).

In this study, objective data (from sensor measurements) and subjective data (from questionnaires) were averaged for every session. Specifically, the following objective and subjective data correspond to each session. The *objective data* are temperature average, humidity average, CO_2_ concentration average, illuminance average, and noise level average. While the *subjective data* are thermal comfort question rate average, IAQ question rate average, visual comfort question rate average, acoustic comfort question rate average, and global comfort question rate average. Age and sex were not considered because the data collected were too homogeneous.

The objective data averages were performed between the collected measurements during the regarded session time intervals, while the subjective data averages were performed between questionnaires conducted at the end of every session. All these averages are thus pre-set as inputs for the MLR technique.

## 4. Results and Discussion

**Figure 11:**
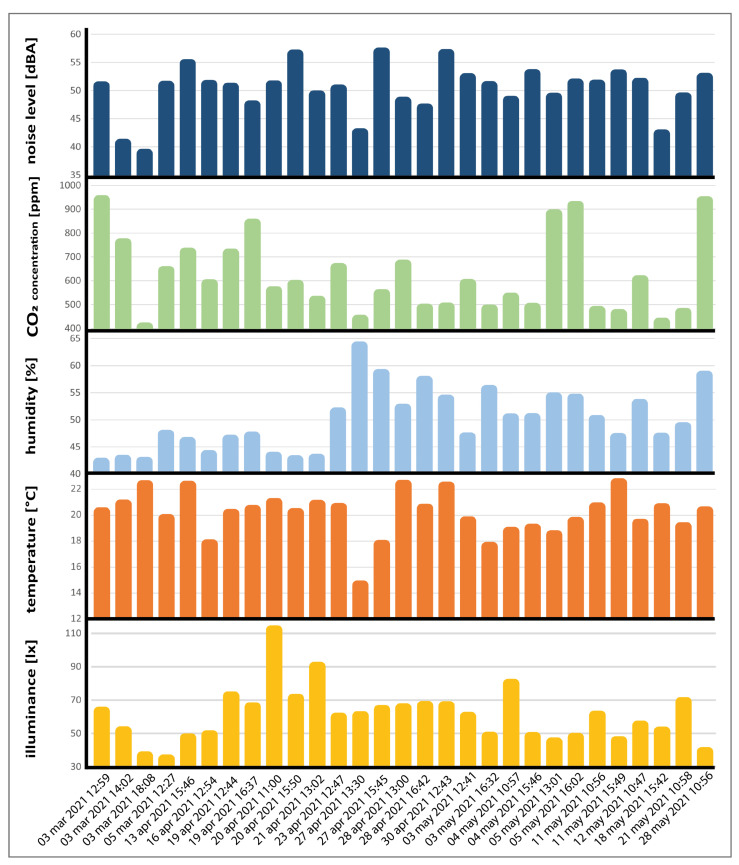
Physical parameter averages per session.

The final goal of this study was to identify a predicted indoor global comfort index (P-IGCI) model, starting from the measured physical quantities, by using this methodology (MLR) and checking whether a better model existed. To achieve this, data were analyzed, and the correlations between the overall comfort (as stated in the questionnaires) with the comfort categories and physical parameters were investigated (in Section 4.1). Then, the most suitable model for calculating a P-IGCI was identified and presented (in Section 4.2). The objective data collected for analysis can be summarized graphically in Figure 11, which shows the averages of the physical parameters per session. The figure consists of five histograms aligned with the X-axis. The X-axis represents the date and time of every session. The Y-axis represents the average measured over the session interval for each physical parameter.

## 4.1. Data Analysis

Firstly, MLR was applied to investigate the relationship between the comfort categories and the global comfort question rate average, treated as the real perceived indoor global comfort index (RP-IGCI). In this case, MLR was used for explanatory purposes, i.e., to understand and weigh the effects of each of the four categories on RP-IGCI reported in the questionnaires. The RP-IGCI stated in the questionnaires was the model’s dependent variable, while the four comfort categories stated in the questionnaires were the independent variables. IBM SPSS Statistics software (version 26) [78] was deployed to perform the MLR. The resulting standardized coefficients (beta) were 0.517 (for thermal comfort), 0.418 (for IAQ), 0.223 (for visual comfort), and 0.246 (for acoustic comfort) with the coefficient of determination R^2^ equal to 0.74. These coefficients are reported on a percentage scale to illustrate the subjective impact of each comfort category on overall comfort:

- Thermal comfort: 37%;
- IAQ: 30%;
- Visual comfort: 16%;
- Acoustic comfort: 17%.

Secondly, MLR was applied to estimate the correlation between the physical parameters and the overall comfort average. Therefore, MLR was used for predictive/estimative purposes, i.e., to identify linear combinations of objective variables to best predict the assumed value by the RP-IGCI. Thus, RP-IGCI is held as the dependent variable, while the four main physical quantities measured (temperature, CO_2_ concentration, illuminance, and noise level) are independent variables.

**Figure 12:**
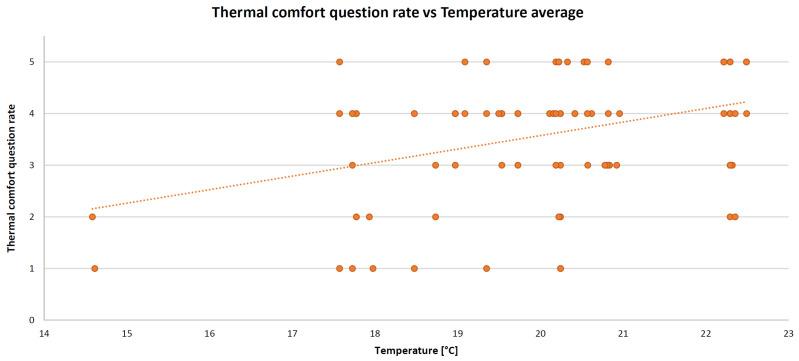
Thermal comfort XY scatter plot and trend line.

**Figure 13:**
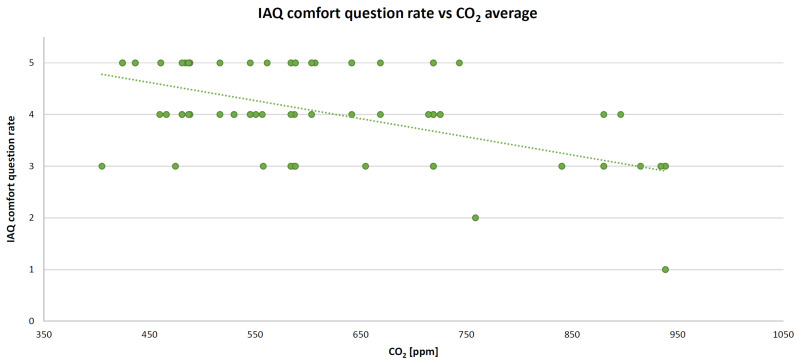
IAQ comfort XY scatter plot and trend line.

**Figure 14:**
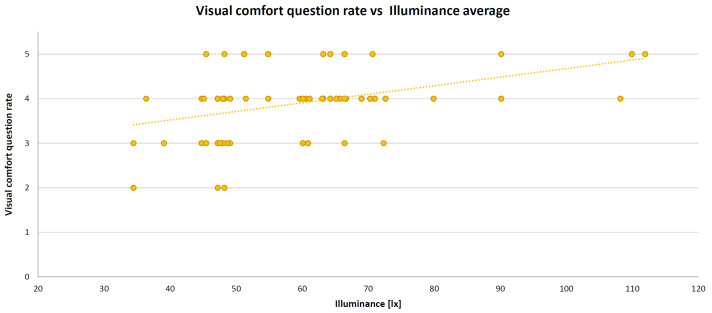
Visual comfort XY scatter plot and trend line.

**Figure 15:**
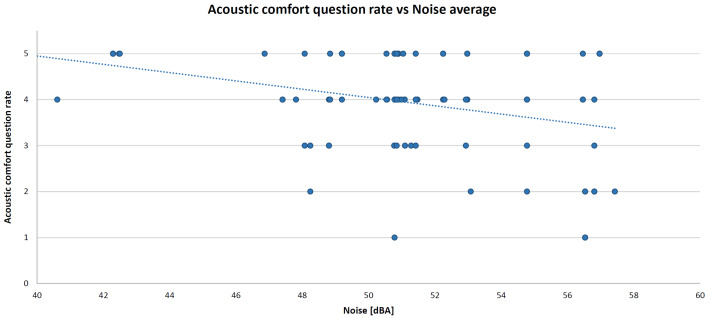
Acoustic comfort XY scatter plot and trend line.

As mentioned above, all of these variables were entered into the algorithm as averages within a lesson (typically one hour). IBM SPSS Statistics software (version 26) was deployed to perform the MLR. The resulting standardized coefficients (beta) are 0.377 (for temperature), −0.538 (for IAQ), −0.035 (for illuminance), and −0.022 (for acoustic comfort) with the coefficient of determination R^2^ = 0.49 and the root mean square error (RMSE) = 0.40 (mean square error (MSE) = 0.16). By adding humidity in the MLR algorithm, the coefficient of determination does not change. For this reason, this parameter was removed from the model; thus, maintaining one physical parameter for each comfort category. The XY scatter plots are presented below. These graphs show the relationship between the comfort category question rate and the corresponding measured objective physical parameter, such as temperature (in Figure 12), CO_2_ concentration (in Figure 13), illuminance (in Figure 14), and noise level (in Figure 15). A high comfort question rate corresponds to a high level of comfort, i.e., 5 equals the “very good” comfort answer from the questionnaire.

For all measured physical quantities, a linear trend line was entered. Obviously, there is a satisfaction value range for temperature and illuminance (unlike noise level and CO_2_ concentration): high/low temperature and too much/too little illuminance generate dissatisfaction. For these reasons, in general, a polynomial (second-degree) trend line would be more suitable. However, in this specific case study, values corresponding to high temperatures (it was not summertime) or glaring light were never detected. Hence, for simplification, a linear relationship is also good.

## 4.2. Model Building and Characterization

This section aims to find a model that outputs a P-IGCI, derived from the measured physical quantities, as close as possible to the RP-IGCI, based on the questionnaires. Both of these indices were calculated over a session. The chosen algorithm was the one that returned the smallest possible RMSE. First, linear regression methods were investigated using SPSS, and the “stepwise” method performed best. Stepwise criteria are a probability of F to enter ≤0.050 and a probability of F to remove ≥0.100. In this way, a model with “CO_2_ concentration” and “temperature” as “variables entered” was achieved. The coefficient of determination R^2^ was 0.46 and RMSE was 0.38 (MSE is 0.14). The “Regression Learner” App in MATLAB (version R2021b) [79] was then used to test the “stepwise” method against other models. Temperature, humidity, CO_2_ concentration, illuminance, and noise level were entered as “predictors”, and RP-IGCI was entered as a “response”. The app tested 19 different algorithms and calculated the corresponding RSME; the values are listed in Table 5. “Stepwise” was confirmed as the best performing model, not only among the linear regression methods, but across all tested methods.

**Figure 16:**
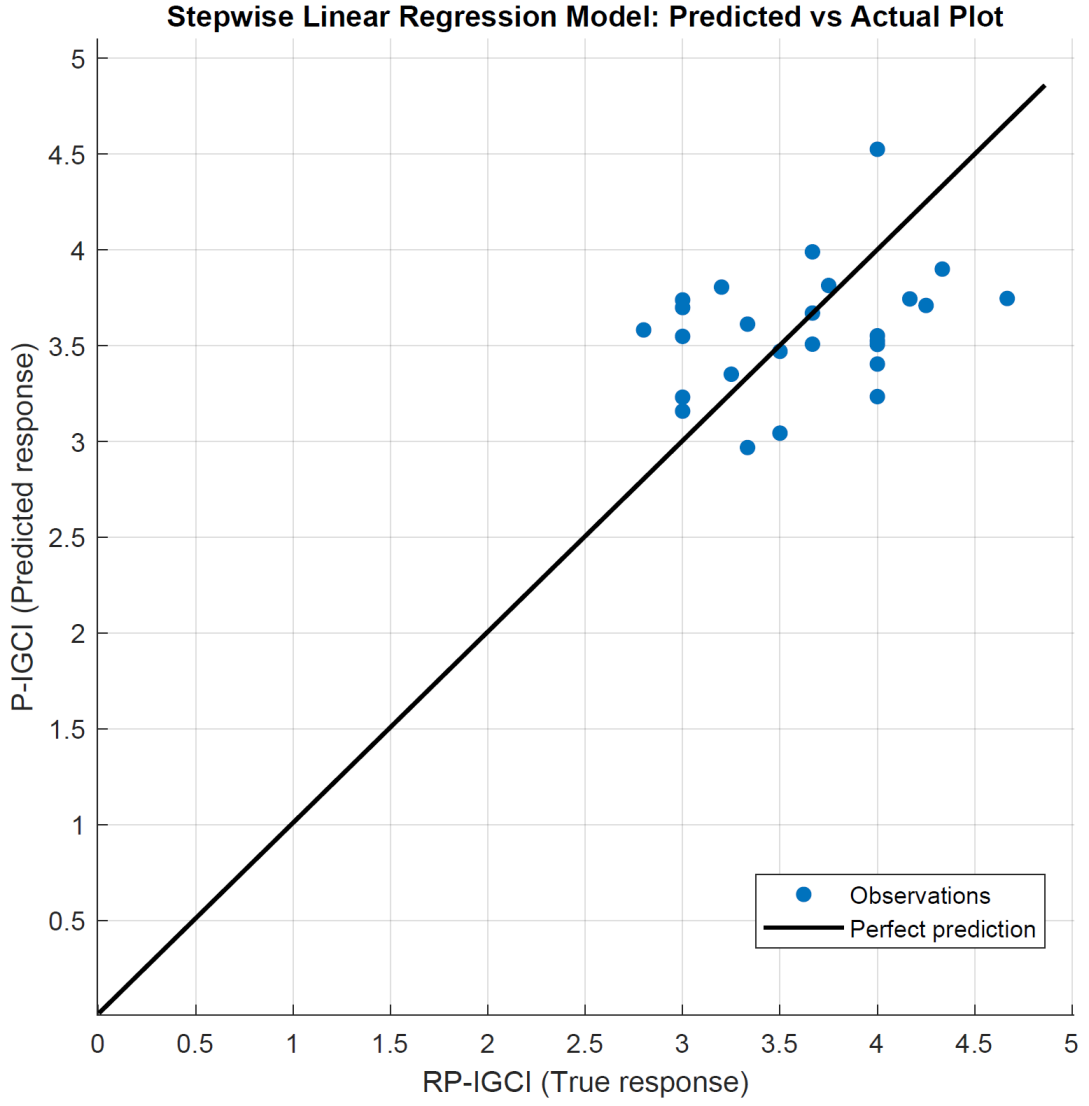
Predicted (P-IGCI) vs. actual (RP-IGCI) plot.

**Figure 17:**
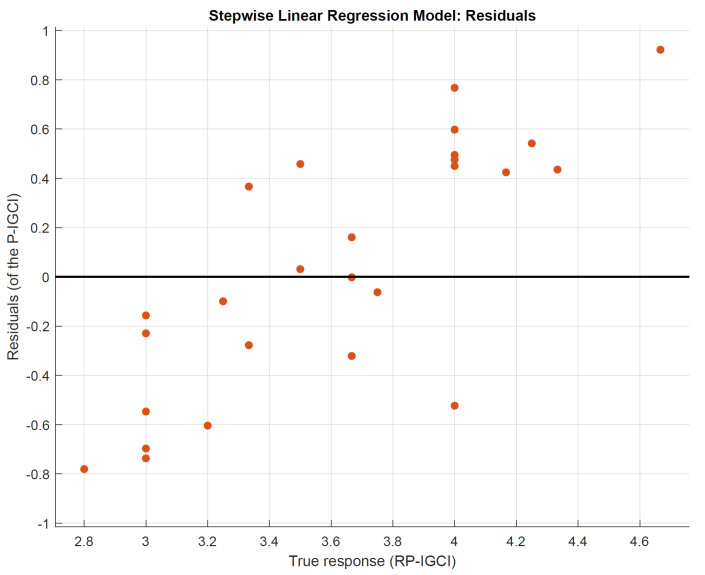
P-IGCI Residuals on true response (RP-IGCI).

**Figure 18:**
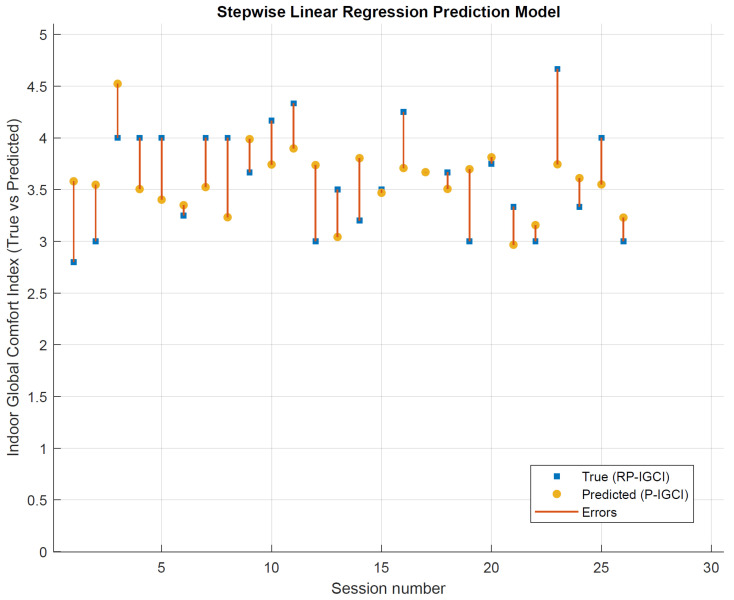
“True (RP-IGCI) vs. predicted (P-IGCI)” error for every session.

In the following, “predicted vs. actual plot” (Figure 16), “residuals plot” (Figure 17), and “response plot” (Figure 18) are illustrated. A high indoor global comfort index (IGCI), either real perceived (RP-IGCI) or predicted (P-IGCI), equals a high level of comfort, i.e., 5 corresponds to the maximum comfort.

In Figure 16, the observation “cloud” around the perfect prediction line is displayed. This figure also reveals that, in general, a good overall comfort was perceived (all points are in the range 2.5–5). In Figure 17, the P-IGCI residuals on the true response (RP-IGCI) are shown. All points are within the −1 and +1 range. This is a very good result since the scale is potentially between −5 and +5. The errors can be better highlighted in Figure 18. The error is minimum when the RP-IGCI point coincides with the P-IGCI point, while it is maximum when the RP-IGCI point is far from the P-IGCI point, i.e., when the red line is longer. The error ranges from a minimum of zero (in session 17, with RP-IGCI = P-IGCI = 3.67) to a maximum of less than 1 (in session 23 with RP-IGCI = 4.67 and P-IGCI = 3.74). The result is positive since it is a maximum error, and the RMSE is equal to 0.38. For example, this means that if a session average comfort of 4 out of 5 is perceived, the model:

- In the worst case, will hardly go to 3 or 5 (±1);
- On average, will return 4.38 or 3.62;
- In the best case, will coincide with 4.00.

This global picture is very good since these are estimates on subjective parameters and a 5-point comfort scale.

## 5. Conclusions

This paper presented a wireless IEQ logger with a DIY philosophy. A simple but comprehensive hardware and software implementation was proposed. The system is designed to monitor all the main types of "comfort" that represent indoor environmental quality, i.e., thermal comfort, indoor air quality (IAQ), visual comfort, and acoustic comfort. The wireless IEQ logger hardware development was possible thanks to the employment of different sensors connected to the Raspberry Pi board. This board operates in an open-source ecosystem. Other hardware involved instruments adopted for the calibration and testing phases of the different sensors. The structure of the questionnaire and, in general, of the entire software, allowed for the organized collection of several objective and subjective data. The specific case study concerns the logger’s use in a university classroom. However, the system implemented is relatively low-cost and can be easily reproduced for monitoring in other classrooms or, more generally, in different indoor environments. The total cost of the IEQ logger was about EUR 150. The price is about the same as a medium quality IEQ logger. The problem is that devices measuring all examined parameters can hardly be found on the market. For example, Netatmo NHC-IT [80] costs about EUR 150 but does not measure illuminance. A professional air quality detector, such as Airthings Wave Plus [81], costs about EUR 250. This device also measures other parameters, such as radon and TVOCs, but does not measure noise and illuminance. Therefore, the wireless IEQ logger is as cheap as a mid-range product for indoor air quality, but it measures all physical parameters related to IEQ categories. Furthermore, it has greater processing capacity, thanks to Raspberry Pi, allowing for upgrade capabilities. Due to the methods adopted, the main objective was achieved (i.e., identifying a P-IGCI model, starting from the measured physical quantities). The MLR technique (between subjective data) allowed to detect the weights of the different comfort categories (thermal comfort 37%, IAQ 30%, visual comfort 16%, acoustic comfort 17%). A first predictive model was found through the MLR technique between objective data and overall subjective comfort (RP-IGCI). Finally, by testing and examining 19 different algorithms, the MLR model with the stepwise method was the best one with the lowest RMSE/MSE. The SPSS (by IBM) and MATLAB (by MathWorks) software were of great help and fundamental importance to achieve these results. Interestingly, the physical quantities excluded from the model identified corresponded to the comfort categories that also subjectively had the lower weights (i.e., visual comfort and acoustic comfort). This result, in general, does not mean that these comfort categories (or the corresponding physical parameters) are useless. The reasons why MLR with the stepwise method discarded these two parameters are: (i) Objective difficulty in measurements (e.g., the voice of the teacher to be distinguished, light varying depending on the position in the room, etc.); (ii) always satisfactory levels: illuminance almost always above 50 lx (as per EN 12464-1) and noise level always below 60 dBA (as per the World Health Organization Community Noise Guidance). In this regard, there are studies [38,82] stating that the level of satisfaction with a comfort type influences the classification of that condition. In other words, the more dissatisfied people are with a condition, the more weight will be given to it; conversely, when people are satisfied with a certain condition, it is considered of less importance [54]. Finally, it is interesting to note that, in several studies on indoor environments [33,34,37,42,82,83], IAQ and thermal comfort are considered the most relevant categories. This fact is even more evident in different Green Building certification schemes [46], in particular by KLIMA [84], LiderA [85], and NABERS [86].

Possible future research could involve the use of artificial intelligence algorithms, such as machine learning techniques, to identify an increasingly accurate predictive model of global comfort. However, these techniques require large amounts of data to be efficient. In this case, more data could be collected by: (i) producing more wireless IEQ loggers, (ii) installing them in more classrooms, in kindergartens, in school rooms (where there are students for a minimum of 6–10 h per day), and (iii) collecting data for a much longer period of time. The most commonly adopted physical parameters for each comfort category were considered in this research. A likely future step could be to improve and certify the IEQ logger with its sensors. For instance, in other contexts, an IEQ logger can be implemented to assess individual comfort categories in accordance with the corresponding international standards. Further investigations should concern even more precise measurements for each comfort category. For thermal comfort, this would involve (i) measuring MRT; (ii) following ISO 7730 [9] and adopting the instrumentation required by ISO 7726 [61]; and (iii) using data from the weather. For visual comfort—it would involve measuring light quality in colour rendering and the homogeneity of light [25]. Acoustic comfort would involve considering echo and acoustic privacy [19]. Eventually, indoor air quality would involve monitoring total volatile organic compounds (TVOCs) [87,88,89,90]. Therefore, the general idea would be to increase the types of sensors to collect and verify if the new parameters have significant impacts on overall comfort.

## Figures and Tables

**Table 1:** Comfort categories and corresponding physical parameters and units.

Comfort Category	Physical Parameter	Unit
Thermal comfort	Air temperature	°C
	Relative humidity	%
Indoor air quality (IAQ)	CO_2_ concentration	ppm
Visual comfort	Illuminance	lx
Acoustic comfort	Noise level	dBA

**Table 2:** Physical parameters and corresponding sensors.

Physical Parameter	Sensor
Air temperature	BME280 sensor on Enviro+ board
Relative humidity	BME280 sensor on Enviro+ board
Illuminance	LTR-559 sensor on Enviro+ board
CO_2_	K-30 sensor
Noise level	USB omnidirectional condenser microphone

**Table 3:** Technical features of the sensors.

Technical Features	BME280	LTR-559	K-30	Microphone
**Interface**	I^2^C			USB 2.0
	(up to 3.4 MHz)	I^2^C (Fast Mode @ 400 kbit/s)	I^2^C	
	SPI		UART	
	(up to 10 MHz)			
**Power** **supply**	1.71–3.6 V	2.4–3.6 V	5–9 V	5 V
			(preferred Operating range)	
**Operating range**	−40…+85 °C		0–10,000 ppm	84 dB (SNR)
	(temperature)	0.01–64 k Lux	(total)	
	0…100%	(6 dynamic range)	0–5000 ppm	
	(rel. humidity)		(within specifications)	
**Accuracy**	±1.0 °C	-	±30 ppm ± 3%	Sensitivity range: within −3 dB (at 1 V)
	(temperature)		(of measured value within specifications)	
	±3%			
	(rel. humidity)			
**Resolution**	0.01 °C (temperature)	16-bit	10 mV	-
	0.008%	(effective resolution)	(8.5 bits in the range 0–4 V)	
	(rel. humidity)			
**Measurement/** **Response Time**	Response Time ($\tau_{63\%}$): 1 s	Integration time:	Response Time (T_1/e_):	
		50 ms	20 s (diffusion time)	Frequency
		Measurement time:	Response Rate:	Response:
		100 ms	2 s	20 Hz–16 KHz
**Dimensions**	2.5×2.5×0.93 mm	2.4×3.9×1.3 mm	∼57×51×14 mm	∼20×5×5 mm
**Other** **specifications**	3 power modes: sleep, normal, forced	- Close to human eye spectral response; - Immunity to IR/UV light source; - Automatically rejects 50/60 Hz lighting flicker.	- Self-diagnostics (complete function check at startup); - ABC (Automatic background calibration).	- Polar pattern: omnidirectional; - Impedance ≤ 2.2 KΩ; - Sensitivity: −30 ± 3 dB.

**Table 4:** Python files and corresponding sensors.

Python Library File	Sensor
temperature.py	BME280 sensor on Enviro+ board
humidity.py	BME280 sensor on Enviro+ board
luminosity.py	LTR-559 sensor on Enviro+ board
co2_level.py	K-30 sensor
noise_level.py	USB omnidirectional condenser microphone

**Table 5:** Tested algorithms and corresponding RMSE/MSE. Stepwise linear has the best RMSE/MSE.

Model	Method	RMSE	MSE
Linear regression	Linear	0.40	0.16
	Interactions linear	0.40	0.16
	Robust linear	0.42	0.18
	**Stepwise Linear**	**0.38**	**0.14**
Regression trees	Fine tree	0.58	0.34
	Medium tree	0.51	0.26
	Coarse tree	0.51	0.26
Support vector machines	Linear SVM	0.47	0.22
	Quadratic SVM	0.47	0.22
	Cubic SVM	0.56	0.31
	Fine Gaussian SVM	0.51	0.26
	Medium Gaussian SVM	0.51	0.26
	Coarse Gaussian SVM	0.47	0.22
Gaussian process	Rational quadratic GPR	0.55	0.30
Regression	Squared exponential GPR	0.53	0.28
	Matérn 5/2 GPR	0.53	0.28
	Exponential GPR	0.52	0.27
Ensembles of trees	Boosted trees	0.51	0.26
	Bagged trees	0.48	0.23

## Data Availability

Not applicable.
